# In vivo turnover and biodistribution of soluble AXL: implications for biomarker development

**DOI:** 10.1038/s41598-024-66665-y

**Published:** 2024-07-12

**Authors:** Olav Tenstad, Eleni Christakou, Linn Hodneland Nilsson, Gro Gausdal, David Micklem, Petri Kursula, James B. Lorens, Rolf K. Reed

**Affiliations:** 1 Department of Biomedicine, University of Bergen, Jonas Lies Vei 91, 5009 Bergen, Norway; 2 BerGenBio ASA, 5009 Bergen, Norway; 3 Faculty of Biochemistry and Molecular Medicine & Biocenter Oulu, University of Oulu, 90014 Oulu, Finland; 4 Centre for Cancer Biomarkers, Norwegian Centre of Excellence, University of Bergen, 5021 Bergen, Norway

**Keywords:** Biomarker, sAXL, Biodistribution, Clearance, Predictive markers, Prognostic markers

## Abstract

Soluble biomarkers are paramount to personalized medicine. However, the in vivo turnover and biodistribution of soluble proteins is seldom characterized. The cleaved extracellular domain of the AXL receptor (sAXL) is a prognostic biomarker in several diseases and a predictive marker of AXL targeting agents. Plasma sAXL reflects a balance between production in tissues with lymphatic transport into the circulation and removal from blood by degradation or excretion. It is unclear how this transport cycle affects plasma sAXL levels that are the metric for biomarker development. Radiolabeled mouse sAxl was monitored after intravenous injection to measure degradation and urinary excretion of sAxl, and after intradermal injection to mimic tissue or tumor production. sAxl was rapidly taken-up and degraded by the liver and kidney cortex. Surprisingly, intact sAxl was detectable in urine, indicating passage through the glomerular filter and a unique sampling opportunity. The structure of sAxl showed an elongated, flexible molecule with a length of 18 nm and a thickness of only 3 nm, allowing passage through the glomerulus and excretion into the urine. Intradermally injected sAxl passed through local and distant lymph nodes, followed by uptake in liver and kidney cortex. Low levels of sAxl were seen in the plasma, consistent with an extended transit time from local tissue to circulation. The rapid plasma clearance of sAxl suggests that steady-state levels in blood will sensitively and dynamically reflect the rate of production of sAxl in the tissues but will be influenced by perturbations of liver and kidney function.

## Introduction

Soluble and circulating proteins are increasingly used as both prognostic and predictive biomarkers for a variety of diseases, driven by personalized medicine initiatives. Soluble factors are readily measured in accessible bodily fluids, primarily plasma, using a growing number of sophisticated methodologies, and levels are correlated with disease outcome. Despite the current interest in soluble biomarkers, very few have been characterized with respect to their fundamental physiological biodistribution and in vivo turnover characteristics that may influence biomarker performance and development.

The AXL receptor tyrosine kinase is an important disease driver and therapeutic target in cancer, fibrosis, as well as infectious and neurodegenerative diseases. The extracellular domain of AXL (sAXL) is cleaved by ADAM-family metalloproteases and released into the interstitial fluid and lymphatic and blood circulation. sAXL levels are suggested to inversely correlate with AXL signaling. Increased sAXL in plasma is associated with inflammatory states and poor prognosis in different diseases^1–6^. Increased sAXL in cerebrospinal fluid is associated with several neurodegenerative diseases. Pharmacodynamic changes in sAXL correlate with AXL kinase inhibition in leukemia patients^7^. However, as with most soluble protein biomarkers, the biodistribution and in vivo characteristics of sAXL are unknown.

sAXL has 345 amino acids and a molecular weight (MW) of 43 kDa, but often appears as 70 kDa due to glycosylation^6^. The apparent MW of sAXL is similar to albumin, the most abundant and most well-studied plasma protein, and albumin may therefore serve as a comparator. Albumin generates more than half of the colloid osmotic pressure in plasma and acts as a generic transport protein for lipophilic substances, including fatty acids. Radioactively labelled albumin has a half-life of about 12 h in the mouse and with a limited passage across the microvascular barrier, including that in the glomerulus. Having entered the extracellular space, proteins the size of albumin will need to be transported back to the circulation via the lymphatics, and in this passage, they are also presented to the lymph nodes before entering the circulation via the thoracic duct. In general, transport from the extracellular and extravascular space directly back to the circulation does not take place for any plasma protein of this molecular weight. Exceptions are endothelial and bone marrow protein sources that access the circulation directly. Hence, in leukemia and some infectious diseases, sAXL likely originates from cells in the bone marrow or spleen.

In contrast, sAXL from a parenchymal cell type or peripheral tumor is transported via lymph to the lymph nodes and might well interact with the cells in the lymph nodes before it is transported further via the larger lymphatic ducts and the thoracic duct to reach the blood stream. The lymph nodes will modify peripheral lymph; as an example, in a human, half of the fluid leaving the circulation across the capillaries is reabsorbed in the lymph nodes and the remaining half transported via the larger lymph vessels to reach the thoracic duct and then enter or reenter the circulation^8^.

In this study, we investigate the physiological turnover and biodistribution of a clinical soluble biomarker and evaluate how these parameters affect biomarker levels.

## Materials and methods

### Animals

Twelve-week-old male BALB/cJRj mice were purchased from Janvier-Labs, France. Animals were housed at the Laboratory Animal Facility of the University of Bergen at a 12 h day/night cycle. All intravenous (i.v.) injections were performed in the tail vein during isoflurane anesthesia (IsoFlo vet 100%, Zoetis, Helsinki, Finland) by using a 0.5 ml syringe (Omnican 50, Braun Melsungen Medical, D-34121 Melsungen, Germany). Isoflurane was administered with a SurgiVet Norvap Service Vaporizer at an induction concentration of 5% and maintenance at 2–2.5% Isoba® vet. Isoflurane under continuous flow of O_2_ (1 l/min). During measurements in the PET scanner, mice were kept warm on a 37 °C heating pad under isoflurane anesthesia. At the end of all experiments, the mice were sacrificed by cervical dislocation while under isoflurane anesthesia.

### Ethical standards

Animal experiments were conducted according to the European Convention for the Protection of Vertebrates Used for Scientific Purposes, Norway in accordance with Directive 2010/63/EU of the European Parliament. Institutional Animal Care and Use Committee (IACUC) protocol numbers were issued by the Norwegian Animal Research Authority to BerGenBio ASA. Studies performed in 2019 have permission ID 8218. Studies performed in 2020 and 2021 have permission ID 17730. The study is reported in accordance with ARRIVE guidelines. A total of 31 mice were used and are reported: 8 mice with i.v. ^18^F or ^18^F-sAxl, 4 mice with intradermal (i.d.) injections ^18^F-sAxl and 19 mice with ^125^I-sAxl.

### Human data

The human data reported are from a study with approval number 2018/2503 at Regional Committees for Medical Research Ethics South East Norway (EUDCRACT 2018-003186-33) (‘Understand consequences of inhibition of receptor-kinase AXL in health and disease’). The serum samples reported here are from the control group and collected from 24 healthy volunteers and analysed with an AXL-specific immunoassay (Myriad RBM, Human CustomMAP HMPC19). All methods were carried out in accordance with relevant guidelines and regulations. Informed consent was obtained from all subjects and/or their legal guardian(s).

### Labelling sAxl with ^18^F

Mouse sAxl was purchased from Sino Biological Inc, China. It was received as lyophilized powder and kept at − 80 °C until the day or day before the experiment when it was labelled with ^18^F as described in Supplemental Material.

### Intravenous and intradermal injections of ^18^F-sAxl

After one purification step, the ^18^F-sAxl was injected i.v. and had less than 5% aggregated high-molecular-weight sAxl both in the injectate and plasma (see “Results” section). Dynamic PET scans were made from the time of i.v. injection to 60 min thereafter during isoflurane anesthesia (n = 8 mice) as described^9,10^. For the i.d. injections, 1 mg sAxl was labelled with ^18^F, resulting in approximately 90% labelling efficiency and less than 5% aggregates. Four mice were injected i.d. with up to 50 μl ^18^F-sAxl during isoflurane anesthesia. A total of 6 injections were made in each mouse (each of the four extremities and each ear). The mice underwent static PET scans in 5 min periods at approximately 1, 2.5, 4 and 5 h. The mice moved freely between the measurements and were sacrificed as described at the end of the 5-h experiment.

### PET analysis of ^18^F-sAxl injections

The integrity of the tracer was confirmed by High Performance Liquid Chromatography (HPLC). PET imaging was performed using a nanoScan PET/CT scanner from Mediso (Mediso Ltd, Budapest, Hungary) and analysed as described^9,10^. Quantitative sampling of ^18^F activity from the reconstructed PET images is described in the Supplemental Material.

### Labelling of sAxl with ^125^I

Labelling of sAxl with ^125^I was performed using an indirect Iodo-Gen® method as described in Supplemental Material.

### Intravenous injection of ^125^I-sAxl

In vivo experiments with ^125^I-sAxl were performed as confirmation experiments using a different method for radioactive labelling. Also, it allows sampling of a wide range of tissues to verify the unexpected turnover observed for sAxl with the ^18^F labelling. A total of 19 mice received i.v. injections of 0.15 ml of ^125^I-labelled sAxl. The mice were anesthetized during injections and moved freely until they were anesthetized again and sacrificed. If available, also urine samples were obtained at end of experiments (10 (n = 4), 20 (n = 4), 40 (n = 3), 60 (n = 4) and 120 (n = 2) min).

### Blood, tissue, and urine samples

Blood samples were drawn intracardially. Details concerning handling of blood and tissue samples are described in Supplemental Material.

### Protein structure prediction

AlphaFold2^11^ models of sAXL were generated at the Google ColabFold site^12^. Visualization and structure analysis were carried out with PyMOL.

### X-ray and light scattering

Mouse recombinant sAxl was from Sino Biological Inc (China) and the extracellular domain of human AXL (corresponding to sAXL) fused to C-terminal Fc was from Evitria (Switzerland). The human sAXL-Fc is a dimer due to the presence of the Fc domain. After initial characterization, we produced a small amount of monomeric human sAXL by proteolytic digestion, followed by protein A purification, and carried out size exclusion coupled with synchrotron small-angle X-ray scattering (SEC-SAXS) for both sAXL-Fc, human sAXL, and mouse sAxl, as described in Supplemental Material.

## Results

**Figure 1:**
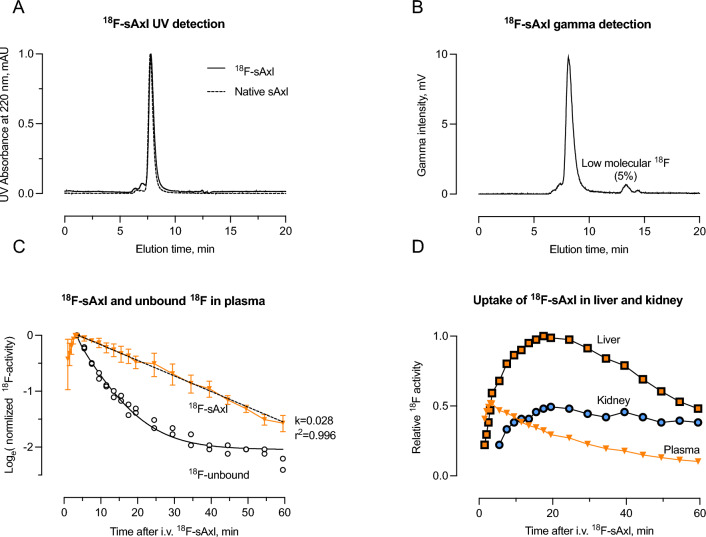
In vivo detection of i.v. ^18^F-sAxl. (**A**) Size exclusion chromatography with UV detection shows overlapping elution patterns of ^18^F-labelled and native sAxl in the injectate. (**B**) Chromatography analysis using gamma-detection confirms the elution patterns observed in (**A**), with approximately 90% of the radioactivity bound to monomeric sAxl. Approximately 5% eluted similar as unbound ^18^F. (**C**) In vivo ^18^F activity sampled from large abdominal blood vessels after i.v. injection of intact ^18^F-sAxl. Data are normalized for comparison across mice (n = 4) by dividing all plasma activities for each mouse by the peak activity observed at 3.5 min. Semi-log linear regression indicated a mono exponential removal of intact ^18^F-sAxl at a rate (k) of 2.8%/min corresponding to a half-life of 25 min. This panel also includes similar measurements in two mice (open circles) injected with unbound ^18^F for comparison. Radioactivity did not accumulate in the kidney cortex and liver, and it decayed similarly to that in plasma in these two mice (data not shown), without the uptake observed for 18F-sAxl (**D**). (**D**) In vivo ^18^F-activity in kidney cortex, liver, and plasma following i.v. injection of ^18^F-sAxl, with normalization to the highest value observed in the liver (n = 4). Error bar represents 1 SD. Quantitative sampling of ^18^F activity from the PET images is explained in detail in the Supplemental Material.

More than 90% of the radioactivity of the purified stock solution of ^18^F-sAxl was bound to monomeric sAxl, with small fractions appearing as high- and low-molecular-weight radioactivity (Fig. 1A,B). After i.v. injection, ^18^F-sAxl was removed from plasma in a mono-exponential manner at a rate of 2.8% per minute, corresponding to a half-life (t_½_) of 25 min (Fig. 1C). Unbound ^18^F was, as expected, removed from plasma faster than ^18^F-sAxl, at a rate of approximately 10% per minute (t_½_ = 7 min), reflecting its rapid diffusion from plasma to the extracellular space, followed by a slower removal through urinary excretion.

^18^F-sAxl was predominantly taken up by the liver and, to a lesser extent, the kidneys (Fig. 1D). This uptake accounted for 50–60% of the injected dose, consistent with the observed removal from plasma. Interestingly, while most activity found in urine represented unbound ^18^F, 6% of the activity recovered in urine was identified as intact ^18^F-sAxl, representing approximately 1% of the injected dose.

**Figure 2:**
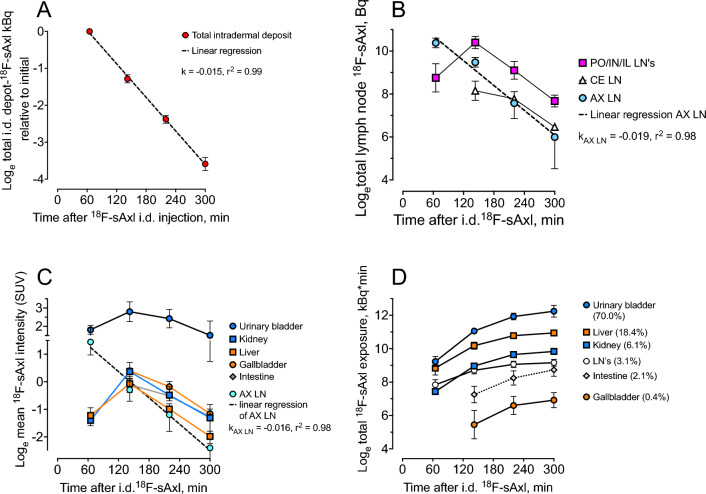
Removal of ^18^F-sAxl from locally injected depots in the ears, front- and hind paws. (**A**) Drainage from the i.d. injection depots. Data points represent all activity from all depots. Slope (k) =  − 0.01514, 95% CI − 0.01587 to − 0.01441. (**B**) Radioactivity in the lymph nodes (LN) draining the three depots: Cervical (CE—open triangles) draining the ears, axillary LN (AX—blue circles) draining the front paws, and draining of the hind paws (red squares) through popliteal (PO), inguinal (IN) LN. The removal rate (k) is similar from the axillary lymph nodes, draining the front paws and local depots, suggesting that these nodes are passive conduits lacking ^18^F-sAxl uptake. Slope (k) =  − 0.01927, 95% CI − 0.02708 to − 0.01146. (**C**) ^18^F concentration normalized to injected dose and body weight concentration (Standardized Uptake Value, SUV) in AX LN, urine, and different organs distant from the injection site. (**D**) Cumulative accumulation of radioactivity as a function of time is measured as the area under the curve (AUC) of ^18^F uptake, quantified as the predicted total radioactivity in kBq within the organ. Numbers in the legends represent the relative distribution of the AUC among the organs, indicating that the liver (18.5%) processes approximately three times more ^18^F-sAxl than the kidney (6.1%). N = 4 mice for all the data points. Error bar represents 1 SD. Slope (k) in Panel (**C**): k =  − 0.016, 95% CI − 0.023 to − 0.009.

Intradermally (i.d.) injected 18F-sAxl was removed at a rate of 1.5% per minute (t½ = 46 min) (Fig. 2A) and detected in local lymph nodes, as well as in the liver, kidneys, and urine, followed by the gallbladder and intestine (Fig. 2B–D). This suggests that sAxl passed through the local lymph nodes and was then processed primarily by the kidneys and liver, releasing metabolites mainly into the systemic circulation and subsequently into the urine, which constitutes the main elimination pathway, and to a lesser extent into the gastrointestinal system. The accumulation in the kidney cortex suggests that intact sAxl is filtered in the glomeruli and taken up by proximal tubular cells where it is degraded. Supplementary Fig. 1 provides additional detailed imaging data supporting these observations. Most of the tracer is excreted via the urine. ^18^F-sAxl was detected in the lymph node nearest the injection site and removed at a similar rate as from the i.d. injection site (Fig. 2B). Peak radioactivity levels in the kidney, liver, and gallbladder were reached at around 140 min post i.d. injection (Fig. 2C). As with i.v. injection, around two-thirds of the ^18^F-sAxl were processed by the liver (Fig. 2D).

The drainage from the front paws is exclusively to axillary lymph nodes, while the drainage from the ears is to the cervical lymph nodes. A more complex pattern is seen for drainage from the hind paws, which is to popliteal lymph nodes around the knee joint, inguinal lymph nodes in the groin as, well as to deep lymph nodes on the posterior abdominal wall. The similar rate of removal of ^18^F-sAxl from the i.d. injection sites and their draining lymph nodes is most likely explained by the ^18^F-sAxl passing through without interacting with or being absorbed by the lymph nodes.

**Figure 3:**
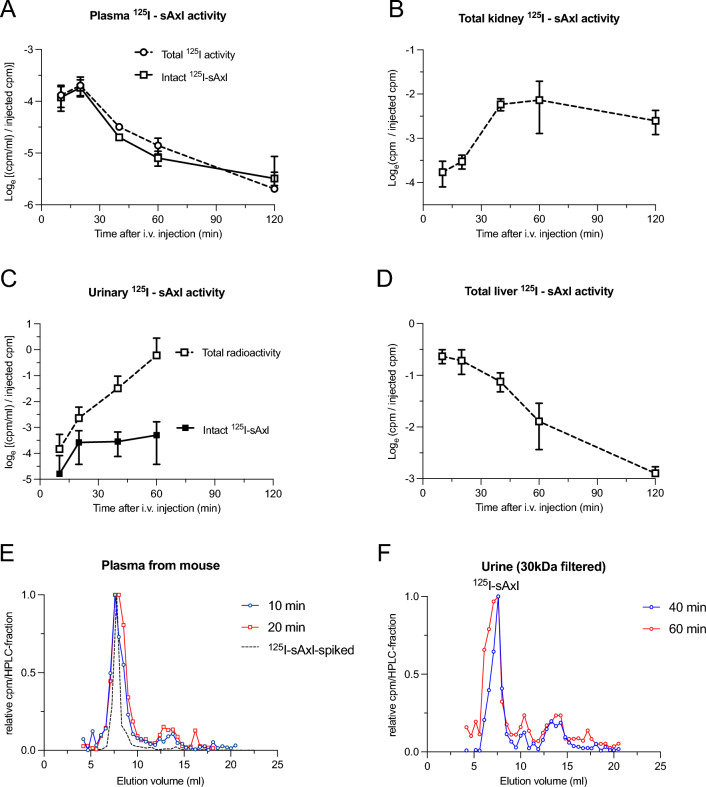
Distribution of ^125^I-sAxl after i.v. injection as a function of time. (**A**) Plasma ^125^I concentration relative to the injected amount of ^125^I-sAxl at different time points after i.v. injection. (**B**) Total kidney radioactivity relative to the injected dose of ^125^I-sAxl. (**C**) Urinary concentration of intact ^125^I-sAxl (filled squares) and unbound ^125^I (open squares) relative to the injected dose of ^125^I-sAxl. (**D**) Total liver radioactivity relative to the injected dose of ^125^I-sAxl. (**E**) Size exclusion HPLC of plasma spiked with ^125^I-sAxl injectate (dashed line) and plasma sampled 10 min (red circles) and 20 min (blue squares) after i.v. injection of ^125^I-sAxl. (**F**) Size exclusion HPLC of concentrated urine (30 kDa spin-filter retentate) sampled 40 min (red circles) and 60 min (blue squares) after i.v. injection of ^125^I-sAxl. Please note the log2 scale on Y-axes in panels (**A–D**).

The distribution of i.v. injected ^125^I-sAxl was analyzed in plasma, kidney, urine, and liver (Fig. 3A–D). The liver showed a rapid uptake, containing about 50% of the injected ^125^I-sAxl within 10–20 min, which reduced to 6% by 120 min (Fig. 3D). Conversely, the kidney exhibited a gradual increase, reaching a plateau near 12% of the dose at 40–60 min, before decreasing to 7% at 120 min (Fig. 3B). The time-integrated ^125^I-sAxl concentration (AUC) being 30% in the kidney and 70% in the liver aligns well with ^18^F-sAxl observations (Fig. 2D). Approximately 80% of the plasma radioactivity, with a half-life of about 40 min, was bound to monomeric sAxl (Fig. 3E), with the rest appearing as unbound ^125^I being excreted into urine. While the total radioactivity excreted into urine increased rapidly, the fraction of intact ^125^I-sAxl in urine relative to the injected amount plateaued after 20 min (Fig. 3C). Thus, the kidney appears to filter intact ^125^I-sAxl, partly excreting it intact into the urine and partly reabsorbing it into proximal tubular cells where it is degraded. Meanwhile, ^125^I-sAxl processed in the liver is degraded, with unbound ^125^I subsequently excreted by the kidney. No sign of split products of ^125^I-sAxl was found in plasma nor in urine (Fig. 3E,F), suggesting complete degradation in the liver and kidney cortex. Intact ^125^I-sAxl in urine was estimated by spin filtration (30 kDa cutoff), and the presence of intact monomeric ^125^I-sAxl was confirmed by size exclusion HPLC (Fig. 3F).

We next characterized the structure of the human sAXL-Fc dimer. Based on SEC-MALS, the AXL-Fc dimer carries ~ 25 kDa of glycans (Supplementary Fig. 2), which corresponds to ~ 15% of its total mass of 165 kDa. The protein MW in the conjugate particle is 140 kDa, which fits with the expected 144 kDa. A synchrotron SAXS experiment carried out on dimeric sAXL-Fc indicated an extended, flexible structure (Supplementary Fig. 2).

**Figure 4:**
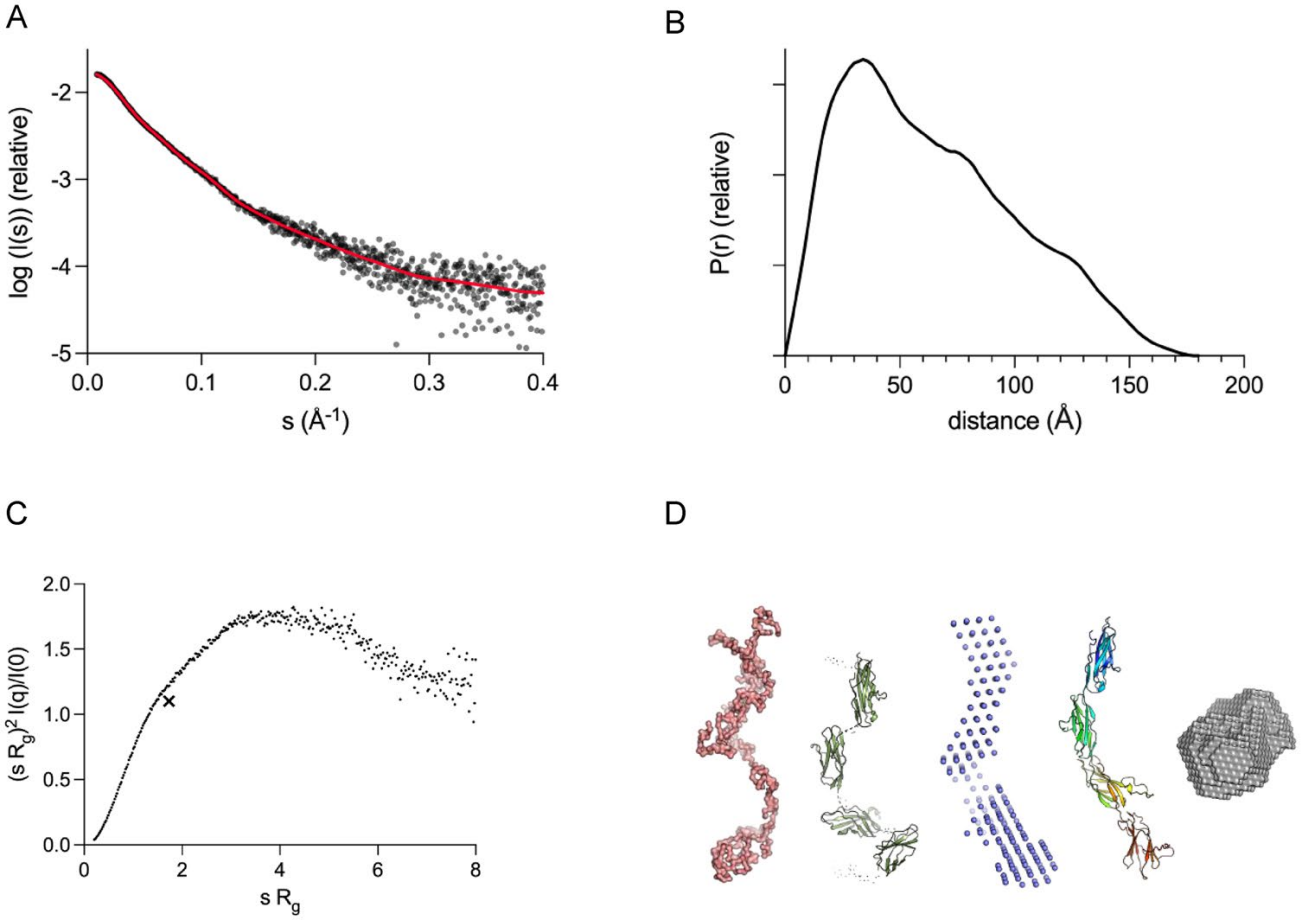
Structural analysis of sAxl. (**A**) SAXS data for mouse sAxl (black) overlaid with the fit of a chain-like ab initio model (red in panel **D**). (**B**) Distance distribution function indicates an elongated multi-domain particle. (**C**) The dimensionless Kratky plot shows a high degree of flexibility. For a globular rigid protein, a single maximum is observed at the position of the cross. (**D**) Models of mouse sAxl. Left to right: ab initio chain-like model based on SAXS (red), a hybrid model based on SAXS (green), a dummy atom-based model based on SAXS (blue), and model from AlphaFold2 (rainbow). To the right, a SAXS-based dummy atom model of globular BSA (gray), with a diameter of 9 nm. Data for human sAXL-Fc and sAXL are shown in Supplementary Figs. 2 and 3.

Next, we studied mouse sAxl and human sAXL with SAXS coupled to molecular modelling (Fig. 4A–D, Supplementary Fig. 3); the data were compared to those for bovine serum albumin (BSA). Notably, sAxl (with glycosylation) and BSA have approximately the same molecular weight, but BSA is a rigid, globular protein (Fig. 4D). sAxl, instead, is highly elongated and flexible (Fig. 4B–D). These differences are evident in both the Kratky plots and the relationship between molecular volume and molecular weight (Fig. 4C, Supplementary Table 1).

Mouse and human sAxl have similar structure and conformation in solution. The four folded domains are arranged like “beads on a string” in the SAXS-based 3D model (Fig. 4D, Supplementary Fig. 3). This results in an elongated, flexible molecule with a maximum length of 18 nm, but a thickness of only 3 nm. The experimental models resemble the corresponding AlphaFold2 model (Fig. 4D). To conclude the analysis, compared to albumin, sAxl is thin and elongated. Hence, as compared to albumin, sAxl is thin and elongated. The globular BSA, with a diameter ~ 9 nm, does not pass through the glomerular filtration system, while earlier work with polymers has shown that extended flexible molecules can orient in the glomerular flow and pass through^13^.

**Figure 5:**
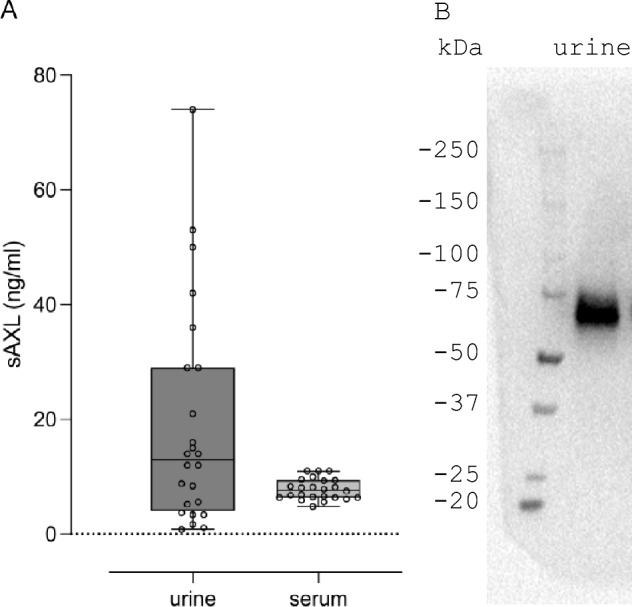
sAXL in human samples. (**A**) Quantification of intact sAXL in urine and plasma of healthy human volunteers (N = 24). (**B**) Western blot of sAXL in human urine.

We verified that sAXL is readily detected in both human plasma and urine by immunoassays, at a median concentration of approximately 10 ng/ml (Fig. 5A). Western blotting with an anti-AXL antibody confirmed that the size of AXL in urine corresponds to the expected size of the intact soluble ectodomain, with an apparent MW of 70 kDa (Fig. 5B).

## Discussion

**Figure 6:**
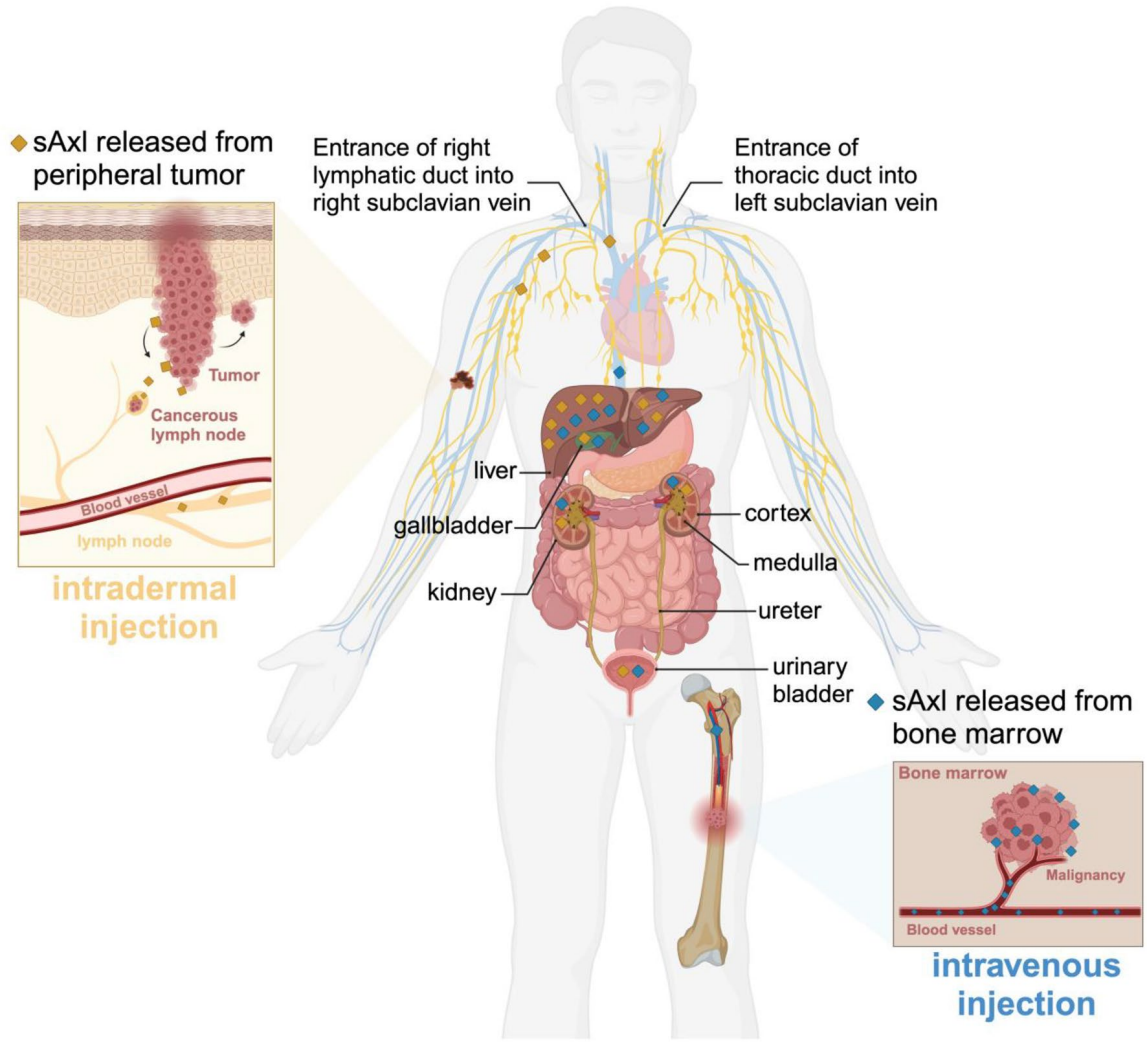
Model of the in vivo turnover and biodistribution of sAXL. Intradermal injection mimics sAXL released from a peripheral tumor, such as in the case of melanoma lymphatic drainage. sAXL accumulates in local and distant lymph nodes, followed by uptake in kidney cortex and liver over time. The stable delivery of intact sAXL and the slow turnover maintains a higher steady-state concentration in the kidney cortex than in the liver (Fig. 2C). In contrast, i.v injection mimics sAXL released from blood vessels or cells in the bone marrow, including myeloid leukemia, that directly enter the circulation, where it is rapidly removed from the circulation by the liver and kidney. Created with BioRender.com.

The in vivo turnover and biodistribution of soluble clinical biomarkers are seldom characterized during biomarker development. Biomarker plasma levels are the result of the balance between production in tissues with lymphatic transport into the circulation and removal from blood by degradation or excretion. Hence, our aim was to investigate the physiological turnover of sAxl to inform clinical biomarker development. The main conclusions of the study are: (1) There is early and dominating uptake of sAxl in the liver with subsequent release; (2) Intact sAxl is present in the urine, providing an unexpected alternative sampling opportunity for this biomarker; (3) sAxl is temporarily retained in lymph nodes when injected intradermally; (4) The half-life of sAxl in the circulation was 25–40 min (Fig. 6). Finally, intact sAxl exhibits an extended molecular structure that allows passage through the glomerular barrier despite its high molecular weight. Additionally, filtered ^18^F-sAxl is partly taken up by the renal cortex, likely through the megalin-cubilin receptor, where it is degraded^14^. The rapid clearance of sAXL from the plasma suggests that steady-state levels detectable in the blood and urine will sensitively and dynamically reflect both the rate of production of sAXL in the tissues but will also be influenced by perturbations of liver and kidney function that affect turnover. The latter is an important consideration when measuring sAXL in patients with metastatic cancer or liver and kidney fibrosis.

The similar molecular weight of sAxl (70 kDa) and albumin belie a dramatic difference in half-life (0.5 vs 12.7 h)^15^. The large difference in in vivo turnover between two proteins of similar hydrodynamic radius reflects both structural differences (globular vs. extended conformation, glycosylation) and perhaps a facilitated sAXL uptake mechanism in the liver.

Proteins the size of sAxl and albumin are expected to drain from the tissues via the lymphatics and return to the circulation via the thoracic duct. However, sAXL produced by tissues such as solid tumors is transiently concentrated in local lymph nodes before returning to the circulation and may be a unique sampling source. In contrast, sAXL from leukemic cells is released directly into the circulation.

These results illustrate how in vivo turnover and biodistribution of a soluble biomarker strongly impact the plasma levels and that disease settings affecting these mechanisms (e.g. liver dysfunction) will independently impact biomarker levels. Intact sAXL is present in the urine, providing an unexpected alternative sampling opportunity for this biomarker. However, further validation is necessary to ensure that urinary sAXL levels accurately correlate with disease status. This validation must also address potential confounding factors such as variations in urine concentration due to hydration status, which can be controlled to some extent by normalizing biomarker levels to creatinine concentration in urine. Additionally, kidney comorbidities must be considered as they may independently affect urinary sAXL level. Moreover, the structural features of biomarker proteins, such as the extended conformation and flexibility of sAXL can affect its glomerular filtration in unexpected ways presenting novel opportunities for urine-based biomarker sampling that can potentially serve to inform on glomerular and kidney function.

### Supplementary Information

Supplementary Information.

## Data Availability

The datasets used and/or analysed during the current study available from the corresponding author on reasonable request.
