# Supplementary material for: In vivo turnover and biodistribution of soluble AXL: implications for biomarker development
Source: Sci Rep. 2024 Jul 12;14:16141. doi: 10.1038/s41598-024-66665-y (PMC11245488; doi:10.1038/s41598-024-66665-y)
Supplement: Supplementary file 1 — Supplementary Information. [file 41598_2024_66665_MOESM1_ESM.pdf]

Supplemental Material for

In vivo turnover and biodistribution of soluble AXL: Implications for biomarker development

Olav Tenstad, Eleni Christakou, Linn Hodneland Nilsson, Gro Gausdal, David Micklem, Petri Kursula, James B. Lorens, Rolf K. Reed

Corresponding author: James B. Lorens, jim.lorens@uib.no

This PDF file includes:

- Supplemental Methods
- Supplemental Figures 1-3
- Supplemental Table 1

Methods

Two different techniques were used for radioactive labelling of sAxl as explained in detail below. For labelling with ^{18}F, [^{18}F]Py-TFP acts as a prosthetic group that facilitates the attachment of the radioactive fluorine-18 to the protein through a lysine residue modification [1]. This method is known for its precision and ability to maintain the biological activity of the labeled protein. In the following text and in the main manuscript, ^{18}F[PyTFP]-sAxl is referred to as ^{18}F-sAxl, and the free tracer, whether it is bound to its prosthetic group or not, is denoted as ^{18}F.

The labeling of sAXL with [^{125}I] was accomplished using Iodogen reagent (1,3,4,6-tetrachloro-3α,6α-diphenylglycouril), which acts as an oxidative agent that facilitates the electrophilic substitution of iodine-125 onto the tyrosine residues of the protein. This method does not involve a prosthetic group in the conventional sense but instead uses a chemical reaction to directly label the protein. The Iodogen method is an indirect labeling technique where the Iodogen reagent remains separate from the protein, thus reducing potential oxidative damage.

Labelling sAxl with ^{18}F

When labeling mouse sAxl, two vials of lyophilized powder (100 µg each) were reconstituted to a concentration of 0.25 mg/ml by adding 0.4 ml of milli-Q water to each vial, in accordance with the manufacturer's recommendations. The s-Axl solution from both vials were then pooled into an Amicon® Ultra 4 mL Filter device (10 kDa molecular weight cutoff) along with 3.2 ml of 0.05 M Na_2HPO_4 in milli-Q water, adjusted to pH 8.5. The mixture was centrifuged to a final volume of 200 µl to remove

potential contaminants and to ensure that the pH was suitable for ^{18}F labeling. ^{18}F was of pharmaceutical grade and obtained from the cyclotron at Haukeland University Hospital, Bergen, Norway on the day of the labelling. Approximately 0.2 mg sAxl was labeled with ^{18}F for PET-imaging by using a similar approach as described by Olberg et al [1]. The purification and integrity assessment of ^{18}F-sAxl were conducted using a PD MidiTrap G-10 gravity column and size-exclusion chromatography HPLC, respectively, with specific columns and conditions as detailed previously [2]. The elution pattern of ^{18}F-sAxl, monitored by in line gamma detection, closely matched that of unlabeled sAxl.

Labelling sAxl with ^{125}I

^{125}I (74 MBq) was obtained from Perkin Elmer (Boston, USA). Batch/ Serial number was I0I2021H/1 as of date 01/20/2021 and was delivered at 74 MBq in a carrier-free, high pH (pH 12-14) formulation of Iodine-125 recommended for Iodogen labeling. A stock solution of sAxl in 200µl Tris Iodination Buffer (25 mM Tris-HCl, pH 7.5, 0.4 M NaCl) was prepared using 200 µg of lyophilized sAxl following a similar procedure as described for ^{18}F labeling. Then, 60 µl of Tris Iodination Buffer and 40 µl of the sAxl stock (approximately 0.8 nmol sAxl) were transferred to the bottom of a 1.8 ml Nunc Cryotube at room temperature. One Pierce™ Pre-Coated Iodination Tube was washed with 1 ml Tris Iodination Buffer, decanted and 100 µl buffer together with 10 µl (37 MBq) carrier free Na ^{125}I (643 GBq/mg) were added to the bottom of the tube. ^{125}I-ions were allowed to activate to ^{125}I+ ions by swirling the iodination tube every 30 s for 6 min at room temperature. Activated ^{125}I was immediately transferred into the tube containing sAxl for incorporation into the tyrosine residues by electrophilic attack of its ortho ring position. The tube was flicked every 30 s for 8 min and the reaction

stopped by adding 50 µl scavenging buffer (10 mg tyrosine/ml in Tris Iodination Buffer, pH 7.4). The solution was mixed and incubated for 5 minutes, with flicking at 1 and 4 minutes, before being transferred to an Amicon® Ultra 15 mL Filter device (10 kDa cutoff). It was then diluted to a total volume of 15 ml with Tris/NaCl/EDTA Buffer (25 mM Tris-HCl, pH 7.5, 0.4 M NaCl, 5 mM EDTA, 0.05% sodium azide) and stored at 4 °C within the device. Shortly before use, the ^{125}I-sAxl solution was concentrated to 200 µl through centrifugation and subsequently washed twice with 14.8 ml of 0.9% saline to remove any unincorporated ^{125}I. Low molecular weight radioactivity accounted for <0.3% of the total activity. The radioactivity was determined in a Gammacounter: Perkin Elmer Wallac Wizzard 1470 auto gamma counter (USA).

PET analysis of ^{18}F-sAxl injections

Dynamic PET scan was performed at the Molecular Imaging Center at University of Bergen on a nanoScan PET/ CT from Mediso (Mediso Ltd, Budapest, Hungary) during isoflurane anesthesia. In vivo experiments were performed immediately following the labelling process and purification. The integrity of the tracer was then confirmed by size-exclusion chromatography HPLC [2].

PET scan acquisition parameters were as follows: Field of view 9.6 cm in the axial direction and 10 cm in the transaxial direction, 1:5 coincidence mode and normal count rate mode [2]. The body temperature was maintained at 37 °C throughout the whole procedure. Reconstruction of the PET data was performed with 3D OSEM and 1:5 coincidence mode, no filtering, attenuation correction (from a helical CT-scan, 50 kVp, 300 ms, 360 projections reconstructed using Ram-Lak filter), decay correction

and normalization of detectors. All images were exported to Image J software (v. 1.47 h, Fiji distribution) [3] for further analysis.

PET experiments were analyzed using InterView Fusion version 3.0.1 (Mediso, Budapest, Hungary), with Volume of Interest (VOI) tools for different organs detailed below and in Supplemental Fig.1.

Blood: Radioactivity was sampled over the large abdominal blood vessels, fitting a VOI-sphere in the center of the lumen across three planes (sagittal, axial and coronal).

Lymph node: ^{18}F-sAxl concentration in lymph nodes was measured using two methods: 1) By calculating the Area Under the Curve (AUC) for mean activity measured as Standardized Uptake Value (SUV) along two orthogonal transects that intersect at the node's peak activity, and 2) By determining the mean activity (SUV) of the lymph node using an isocount 3D Region of Interest tool. The average ^{18}F-sAxl activity (SUV) was then calculated as the mean of these two results. The total radioactivity (in Bq) was estimated based on the content within the segmented lymph node.

Intradermal (i.d.) depot, intestine, gallbladder, and urinary bladder: The calculation of average ^{18}F-sAxl intensity and total radioactivity for the six i.d. depots, as well as for the gallbladder, urinary bladder, and intestine, followed the same methodology applied to the local lymph nodes. Specifically, the average intensity for the intestine was also calculated as described above, with its total activity determined through iterative use of the isocount 3D tool. However, it is important to note the potential error due to loss of radioactivity from the gallbladder, urinary bladder, and intestine when the animals are freely moving between the PET scans.

Kidney cortex and liver: ^{18}F-sAxl intensity in the kidney cortex and liver was measured along two orthogonal transects and within two spherical VOIs of 1 mm and 3 mm in diameter, respectively, as illustrated in Supplementary Fig. 1. The mean intensity was calculated as described above. The total content of ^{18}F-sAxl (in kBq) was estimated from the mean intensity and the predicted total organ mass based on body weight, using data from Marxfeld et al [4]. The mass of the kidney cortex was assumed to be 70% of the total kidney mass.

Blood, tissue, and urine samples

Blood samples were drawn intracardially using a 0.5 ml syringe (Omnican 50, B Braun Melsungen Medical, D-34121Melsungen, Germany). The blood was then centrifuged (Eppendorf Centrifuge 5417 C, Hamburg, Germany) at 1,500 G for 10 min to obtain serum and about 40 µl was then pipetted into pre-weighed vials which were then reweighed to obtain the exact amount of serum in the vial.

Tissue samples. After determination of tissue and organ weight to the nearest 0.1 mg, radioactivity was determined. Extravasation was calculated as ml plasma per g tissue by dividing (cpm per g tissue) by (cpm per ml serum). The liver and each kidney were taken out in total which allowed determination of their amount of the injected dose. Urine samples were obtained, when possible, at the end of the experiment by suprapubic puncture. Tissue and serum samples were weighed on a DeltaRange (Mettler Toledo, Spain) to the nearest 0.1 mg. The syringe for injection and blood withdrawal was a 0.5 ml syringe (Omnican 50, B Braun Melsungen Medical, D-34121 Melsungen, Germany).

Multi-angle light scattering

Multi-angle light scattering coupled to SEC (SEC-MALS) was carried out for sAXL-Fc at the core facility for Biophysics, Structural Biology, and Screening (BiSS) at the University of Bergen. Chromatography was performed using Äkta Purifier (GE Healthcare) and a Superdex 200 Increase 10/300GL column (GE Healthcare) in a running buffer containing 10 mM histidine, 150 mM NaCl, pH 6. A sample of 250 µg of sAXL-Fc was injected into the column at an isocratic flow of 0.4 mg/ml and light scattering recorded using Wyatt miniDAWN TREOS detector. The UV Absorbance recorded at 280 nm was used as a concentration source using the extinction coefficient of sAXL-Fc (0.1% Abs 1.3, calculated in ProtParam using the protein sequence). Conjugate analysis was performed in the ASTRA software (Wyatt) to obtain the molecular weight of sAXL-Fc and glycans.

SAXS Data Acquisition and Data analysis

Aliquots of sAXL-Fc (3.6 mg/ml) in phosphate buffered saline (PBS) containing 100 mM arginine were kept at -80 °C until use. Monomeric sAXL was prepared in small scale as follows. Immobilized papain (ThermoFisher) was equilibrated in digestion buffer (PBS, 20 mM cysteine, 0.5 M EDTA, pH 7) following manufacturer's instructions. sAXL-Fc was mixed with the equilibrated papain (5 mg/ml papain slurry), and the digestion mix was incubated shaking at 4 °C overnight. The reaction was stopped by centrifugation and filtration to remove immobilized papain. sAXL was separated from Fc fragments and intact sAXL-Fc by loading the digestion mix into a gravity flow column with 1 ml MabSelect Xtra (GE Healthcare) resin pre-equilibrated

with PBS, pH 7. The resin was washed with 5 column volumes of PBS, and sAXL was collected in the flowthrough and initial wash fractions. The Fc fragments and intact sAXL-Fc were eluted with 50 mM sodium acetate, pH 3.

SEC-SAXS data for sAXL-Fc (3.6 mg/ml), human sAXL (0.4 mg/ml), mouse sAxl (0.6 mg/ml) and monomeric BSA (1.9 mg/ml) were collected on the SWING beamline at Synchrotron Soleil (Paris, France) [5]. During the measurement, the sample was run through an Agilent Advance BioSEC 300Å column in a buffer containing 10 mM histidine, 150 mM NaCl, pH 6 (for sAXL-Fc) or 20 mM Tris, 150 mM NaCl, pH 7 (for sAXL, sAxl and BSA) at 4 °C. Initial processing was performed on the beamline using FOXTROT. The sAxl and BSA data were collected on the same day, using the same column, for best comparison.

Data were further processed in CHROMIXS [6]. All SAXS data were analyzed using the ATSAS software package [7]. Specifically, PRIMUS [8] was used for data analysis, GNOM [9] for distance distribution, and GASBOR [10] and CORAL [11] for 3D modeling. Dummy atom models were built using DAMMIN [12] and dummy residue models with GASBOR. Fitting of the sAXL AlphaFold2 model to the SAXS data was done using CRYSOL [13].

Figures

171

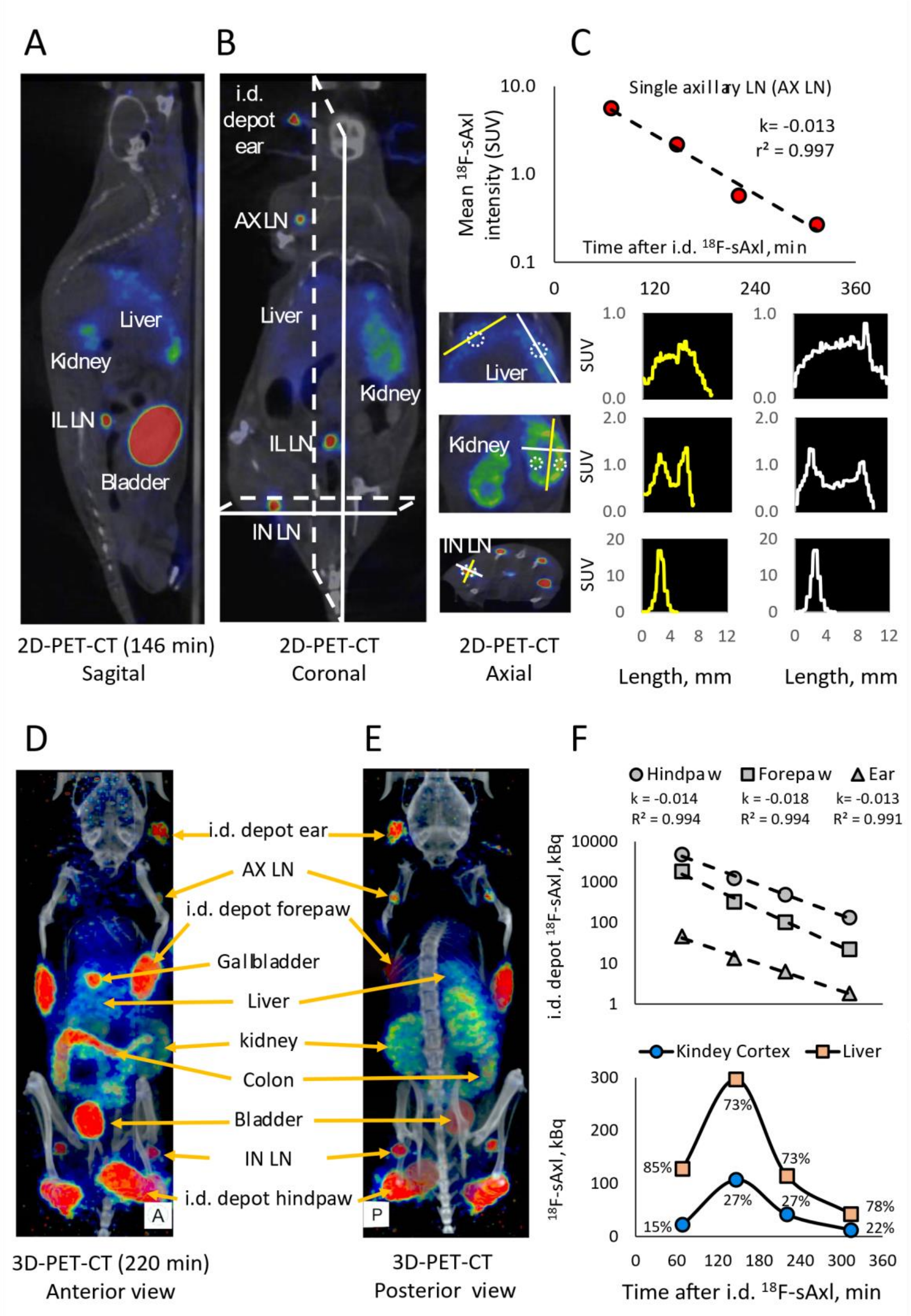

A
B
C
i.d. depot ear
AX LN
Liver
Kidney
IL LN
Bladder
IN LN
Single axillary LN (AX LN)
k= -0.013
r² = 0.997
Mean ¹⁸F-sAxl intensity (SUV)
Time after i.d. ¹⁸F-sAxl, min
SUV
2D-PET-CT (146 min)
Sagital
2D-PET-CT
Coronal
2D-PET-CT
Axial
Length, mm
Length, mm
D
E
F
i.d. depot ear
AX LN
i.d. depot forepaw
Gallbladder
Liver
kidney
Colon
Bladder
IN LN
i.d. depot hindpaw
Hindpaw
Forepaw
Ear
k = -0.014
R² = 0.994
k = -0.018
R² = 0.994
k= -0.013
R² = 0.991
i.d. depot ¹⁸F-sAxl, kBq
Kindey Cortex
Liver
¹⁸F-sAxl, kBq
85%
73%
73%
78%
15%
27%
27%
22%
Time after i.d. ¹⁸F-sAxl, min
3D-PET-CT (220 min)
Anterior view
3D-PET-CT
Posterior view

Supplemental Figure 1. Distribution of 18F-sAxl 146 and 220 min post i.d. injection. (A) is a sagittal section and **(B)** is a coronal section demonstration of the position of the sagital section in Panel A and the axial section in the lower panel of Panel C. IN and AX are inguinal and axillary lymph nodes (LN), respectively. **(C)** Demonstrates how the VOI is determined in liver, kidney and lymph nodes together with radioactivity profiles along the two planes set for each organ. The lower Panel in C demonstrates four inguinal lymph nodes. In the left lymph node, which is also seen in the coronal section in Panel B, the *Volume of Interest* (VOI) where the total radioactivity (in Bequerel, Bq) as well as Standardized Uptake Value (SUV) is measured. Also, two sections (perpendicular to each other) are also shown and the activity in these two profiles is shown in the rightmost panels. Above the lymph nodes are shown coronal sections from liver and kidney not covered by the section in Panel B. (**D,E**) demonstrate anterior and posterior views of a 3D-reconstrction of a mouse documenting intensity of ^{18}F-sAxl in the different organs. **(F)** Radioactivity in the intradermal (i.d.) deposit as well as radioactivity in liver and kidney as a function of time. The numbers for kidney and liver are the fractions of radioactivity in these two organs. Color coding in all panels: Red depicts highest radioactivity, blue the lowest and yellow and green intermediate between these two.

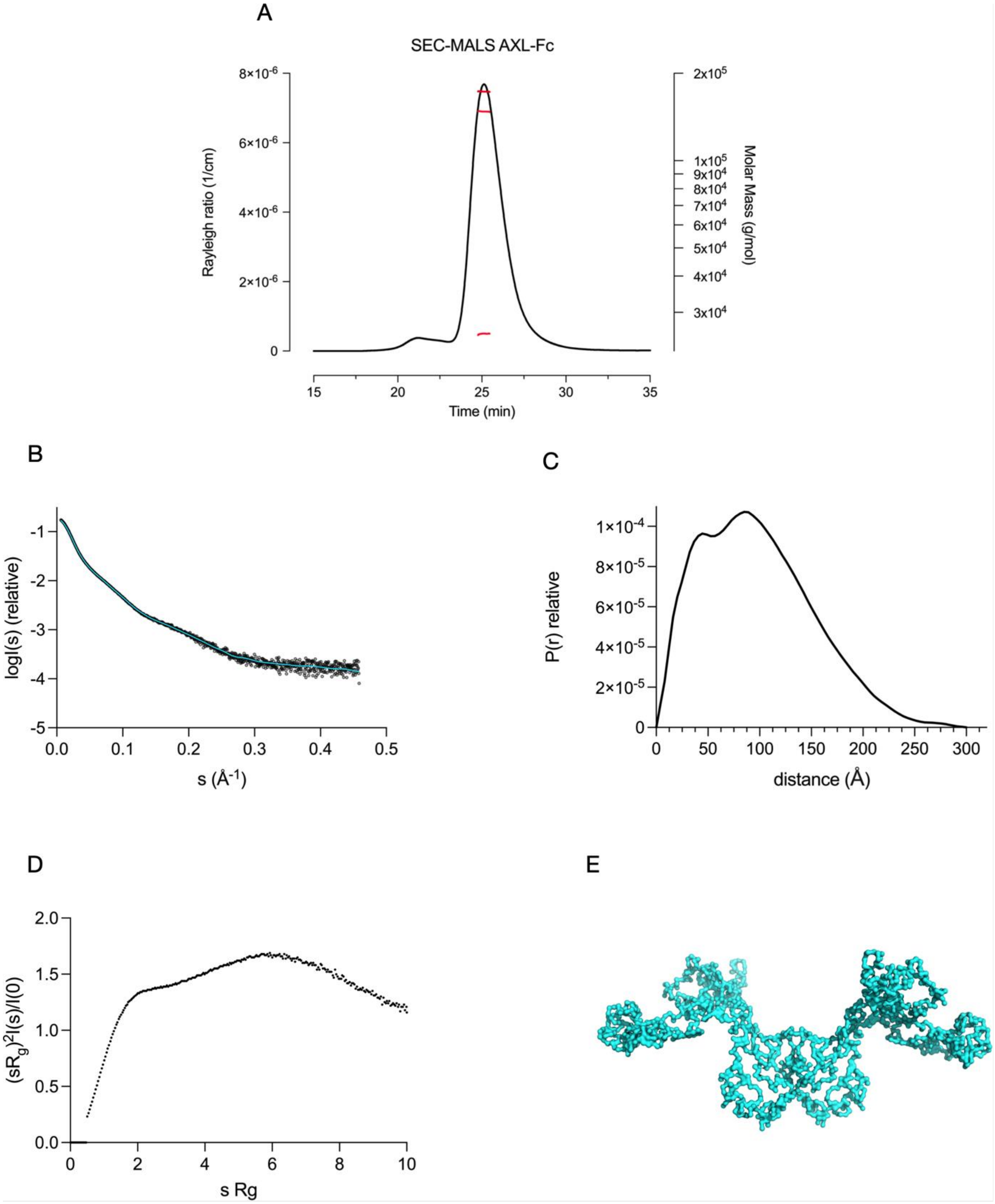

Supplemental Figure 2. **Structural analysis of human sAXL-Fc in solution.** (**A**) SEC-MALS with conjugate analysis to determine the level of glycosylation and the absolute MW. Molecular weights within the peak are shown by red lines (from top to bottom: full MW, protein

MW, glycan MW). (**B**) SAXS scattering data. The fit of the GASBOR model in Panel E is plotted over the measurement data with a cyan line. **(C)** Distance distribution. **(D)** Dimensionless Kratky plot. **(E)** GASBOR *ab initio* model of sAXL-Fc based on the SAXS data. Flexible sAXL molecules extend away from the Fc dimer in the middle.

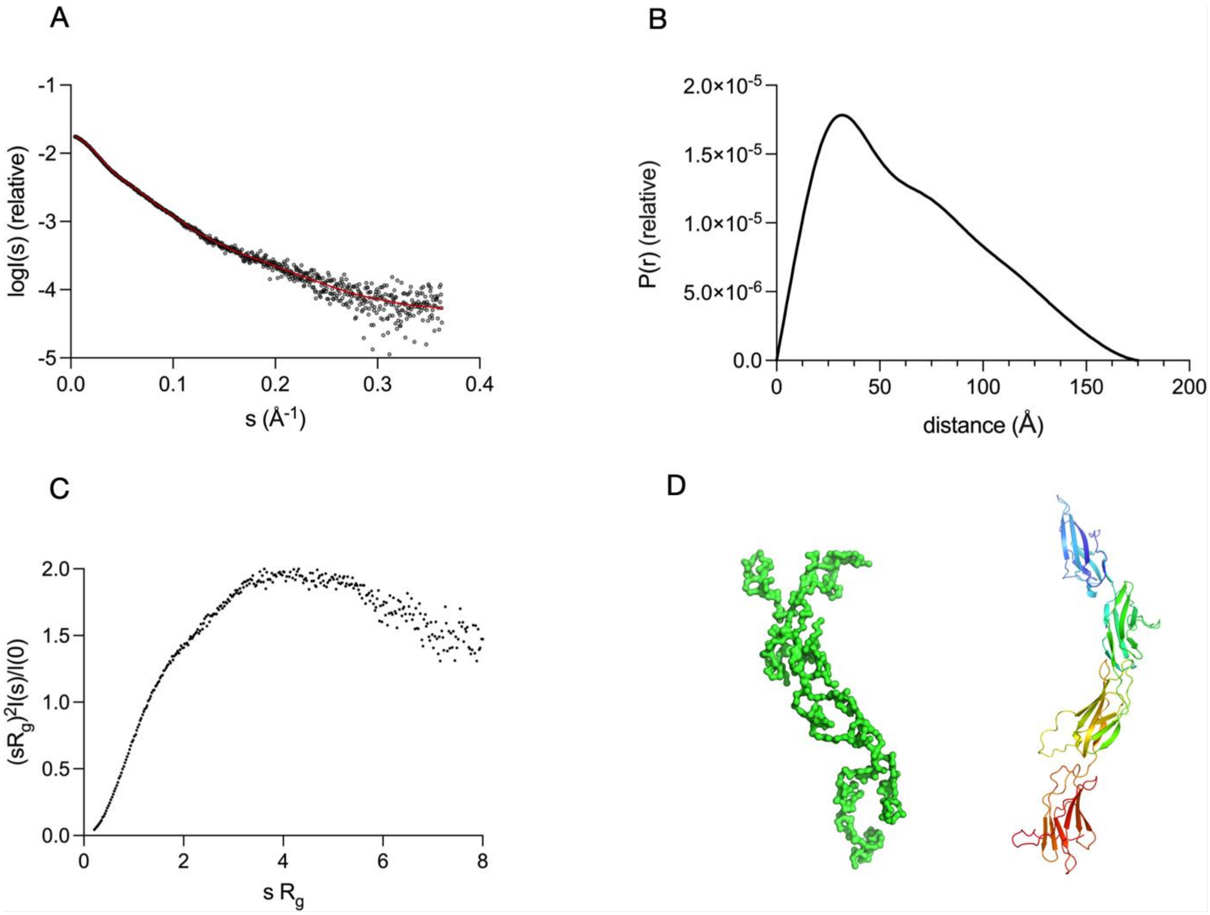

Supplemental Figure 3. Structural analysis of monomeric human sAXL in solution. (A) SAXS data. The fit of the GASBOR model in panel D is shown with a red line. **(B)**. Distance distribution. **(C)** Dimensionless Kratky plot. **(D)** Chain-like model of sAXL based on SAXS data (left) and an AlphaFold2 model of the same construct (right).

<u>Tables</u>

sample	MW based on sequence (kDa)	R_g (nm)	D_{max} (nm)	Volume (nm^3)	MW estimate from SAXS (kDa)
sAXL-Fc dimer	144	7.64	30.0	502	243
human sAXL	43 *	4.87	17.7	137	63.9
mouse sAxl	48 *	4.89	18.0	141	59.5
BSA	67	2.87	9.0	117	65

* without glycosylation

Supplemental Table 1. Molecular size and shape of sAxl based on SAXS. The volume is based on *ab initio* bead-like modelling of the structure in DAMMIN, and the MW estimate represents the Bayesian estimate based on raw SAXS data. R_g was obtained from the Guinier plot and D_{max} from the distance distribution function.

References

1 Olberg, D. E. *et al.* One step radiosynthesis of 6-[(18)F]fluoronicotinic acid 2,3,5,6-tetrafluorophenyl ester ([(18)F]F-Py-TFP): a new prosthetic group for efficient labeling of biomolecules with fluorine-18. *J Med Chem* **53**, 1732-1740, doi:10.1021/jm9015813 (2010).

2 Muslimovic, A. *et al.* Novel clearance of muscle proteins by muscle cells. *Eur J Cell Biol* **99**, 151127, doi:10.1016/j.ejcb.2020.151127 (2020).

3 Schindelin, J. *et al.* Fiji: an open-source platform for biological-image analysis. *Nat Methods* **9**, 676-682, doi:10.1038/nmeth.2019 (2012).

4 Marxfeld, H. A., Kuttler, K., Dammann, M., Groters, S. & van Ravenzwaay, B. Body and organ weight data in 28-day toxicological studies in two mouse strains. *Data Brief* **27**, 104632, doi:10.1016/j.dib.2019.104632 (2019).

5 Thureau, A., Roblin, P. & Perez, J. BioSAXS on the SWING beamline at Synchrotron SOLEIL. *Journal of Applied Crystallography* **54**, 1698-1710, doi:10.1107/S1600576721008736 (2021).

6 Panjkovich, A. & Svergun, D. I. CHROMIXS: automatic and interactive analysis of chromatography-coupled small-angle X-ray scattering data. *Bioinformatics* **34**, 1944-1946, doi:10.1093/bioinformatics/btx846 (2018).

7 Manalastas-Cantos, K. *et al.* ATSAS 3.0: expanded functionality and new tools for small-angle scattering data analysis. *J Appl Crystallogr* **54**, 343-355, doi:10.1107/S1600576720013412 (2021).

8 Konarev, P. V., Volkov, V. V., Sokolova, A. V., Koch, M. H. J. & Svergun, D. I. PRIMUS: a Windows PC-based system for small-angle scattering data analysis. *Journal of Applied Crystallography* **36**, 1277-1282, doi:10.1107/S0021889803012779 (2003).

9 Svergun, D. I. Determination of the regularization parameter in indirect-transform methods using perceptual criteria. *Journal of Applied Crystallography* **25**, 495-503, doi:10.1107/S0021889892001663 (1992).

10 Svergun, D. I., Petoukhov, M. V. & M.H.J., K. Determination of Domain Structure of Proteins from X-Ray Solution Scattering. *Biophysical Journal* **80**, 2946-2953, doi:10.1016/S0006-3495(01)76260-1 (2001).

11 Petoukhov, M. V. *et al.* New developments in the ATSAS program package for small-angle scattering data analysis. *J Appl Crystallogr* **45**, 342-350, doi:10.1107/S0021889812007662 (2012).

12 Svergun, D. I. Restoring low resolution structure of biological macromolecules from solution scattering using simulated annealing. *Biophys J* **76**, 2879-2886, doi:10.1016/S0006-3495(99)77443-6 (1999).

13 Franke, D. *et al.* ATSAS 2.8: a comprehensive data analysis suite for small-angle scattering from macromolecular solutions. *J Appl Crystallogr* **50**, 1212-1225, doi:10.1107/S1600576717007786 (2017).
